# Intralesional gene expression profile of JAK-STAT signaling pathway and associated cytokines in *Leishmania tropica-*infected patients

**DOI:** 10.3389/fimmu.2024.1436029

**Published:** 2024-09-19

**Authors:** Shima Hadifar, Nasrin Masoudzadeh, Hossein Heydari, Vahid Mashayekhi Goyonlo, Mohammadali Kerachian, Maryam Daneshpazhooh, Ali Sadeghnia, Nasim Tootoonchi, Reza Erfanian Salim, Sima Rafati, Ali M. Harandi

**Affiliations:** ^1^ Department of Immunotherapy and Leishmania Vaccine Research, Pasteur Institute of Iran, Tehran, Iran; ^2^ Cutaneous Leishmaniasis Research Center, Mashhad University of Medical Sciences, Mashhad, Iran; ^3^ Autoimmune Bullous Disease Research Center, Tehran University of Medical Sciences, Tehran, Iran; ^4^ School of Medicine, Razi Hospital, Tehran, Iran; ^5^ Noor Eye Hospital, Tehran, Iran; ^6^ Department of Microbiology and Immunology, Institute of Biomedicine, Sahlgrenska Academy, University of Gothenburg, Gothenburg, Sweden

**Keywords:** *Leishmania tropica*, cutaneous leishmaniasis, JAK-STAT signaling pathway, cytokines, immune checkpoints

## Abstract

**Background:**

The JAK-STAT signaling pathway is a central cascade of signal transduction for the myriad of cytokines in which dysregulation has been implicated in progression of inflammatory and infectious diseases. However, the involvement of this pathway in human cutaneous leishmaniasis (CL) due to *Leishmania* (*L.*) tropica warrants further investigation.

**Methods:**

This study sought to investigate differential gene expression of several cytokines and their associated *jak-stat* genes in the lesions of *L. tropica*-infected patients byquantitative Real-Time PCR. Further, the expression of five inhibitory immune checkpoint genes was evaluated.

**Results:**

Results showed that the gene expression levelsof both Th1 (*ifng*, *il12*, *il23*) and Th2 (*il4*, *il10*) types cytokines were increased in the lesion of studied patients. Further, elevated expression levels of *il35*, *il21*, *il27* and *il24* genes were detected in the lesions of CL patients. Notably, the expression of the majority of genes involved in JAK/STAT signaling pathway as well as checkpoint genes including *pdl1*, *ctla4* and their corresponding receptors was increased.

**Conclusion:**

Our finding revealed dysregulation of cytokines and related *jak-stat* genes in the lesion of CL patients. These results highlight the need for further exploration of the functional importance of these genes in the pathogenesis of, and immunity to, CL.

## 1 Introduction

Cutaneous leishmaniasis (CL) is a complex skin infection caused by over 20 different *Leishmania* species that are transmitted through the bite of infected sandflies. CL is endemic in more than 90 countries, with most infections occurring in South and Central America, the Mediterranean Basin, and regions ranging from the Middle East to Central Asia (1). In the Old World, the etiologic agents of CL include *Leishmania* (*L.*) *major*, *L. tropica*, and *L*. *aethiopica*, and also less commonly in the Mediterranean Basin, *L. infantum* and *L. donovani* (2). In terms of clinical manifestations, there are two main types of CL: zoonotic CL (ZCL) caused by *L. major* (rural or wet type lesion) and anthroponotic CL (ACL) caused by *L. tropica* (urban or dry type lesion) (3). The *L. tropica* that causes ACL is restricted to humans and has shown drug resistance, so treating CL patients successfully is challenging. Further, unresponsive chronic patients such as recurrence cases (recidivans) serve as a reservoir of infection for other individuals since a human is an isolated reservoir host (4, 5). Nevertheless, only a limited number of human research have concentrated on understanding immune response mechanisms involved in *L. tropica* infection (6–8).

Several studies have evaluated the expression of key cytokines related to intralesional and systemic immune response during the acute and chronic stages of CL infection caused by *L. tropica* (8–10). Cytokine gene expression study in CL lesions has revealed higher levels of *ifng, il10, tnfα, il1β, il8, il4, mcp1*, and *inos*, indicating CL is caused by an exaggerated and inadequately controlled T helper 1 (Th1) immune response (8). Furthermore, in chronic ACL patients, high levels of IFN-γ, IL-5, and IL-13 imply a mixed Th1/Th2 response, whereas, in acute patients, high levels of IFN-γ and low levels of IL-5 and IL-13 suggested a predominantly Th1 response (9). The differentiation of Th cells is regulated by transcription factors such as Tbet and GATA3, which can, in turn, convert naïve CD4^+^ T cells into Th1 and Th2 subsets (11). The biological effect of most Th1/Th2 associated cytokines is mediated by Janus kinase/signal transducer and activator of transcription (JAK/STAT) signaling pathway, which is reported to have an important function in promoting the host immune response against various diseases (12).

The human JAKs family known as non-receptor tyrosine protein kinases contains four members, JAK1, JAK2, JAK3, and TY2, which transmit regulatory signals of cytokine and recruit one or more STAT proteins. The human STATs family, as transcription factors, contains six STATs, STAT1-6, activated by tyrosine phosphorylation by the JAKs family (13). They are essential for modulating signaling that occurs downstream of cytokine receptors (13, 14). Despite the progress on cytokines’ critical roles in shaping the host immune response to *Leishmania* infection, the role of the JAK/STAT pathway in mediating biological activities of cytokines is rather understudied in human CL, particularly in CL due to *L. tropica* (12). Additionally, in infectious diseases such as CL, immune checkpoints have been demonstrated to have a significant impact on disease outcomes by regulating T cell effector activity (15). Inhibitory immune checkpoint molecules, or inhibitory receptors, are expressed in numerous immune cells, leading to exhausted T-cells and promoting pathogenesis (16). Our recent study on the transcriptome of ulcerative (UCL) and non-ulcerative (NUCL) patients infected with *L. tropica* highlighted the enrichment of “IFNs signaling”, “TCR complex” and “FcγR-dependent phagocytosis pathways (17).

The present study aimed to shed light on *jak*/*stat*-related gene expression patterns in skin biopsy samples collected from CL patients infected with *L. tropica* by quantitative Real-Time PCR (qRT-PCR). Further, differential gene expression of Th1 and Th2-related cytokines, transcription factors, and inhibitory checkpoints were investigated. Our results pinpoint the intralesional profile between *jak*/*stat*-related genes and related inflammatory cytokines that are involved in the pathogenicity of *L. tropica-*infected patients.

## 2 Materials and methods

### 2.1 Ethics approval and informed consent

This study was approved by the Research Ethics Committee of the Pasteur Institute of Iran (IR.PII.REC.1400.022), and study protocols were performed in compliance with the relevant ethical regulations. Written informed consent was obtained from all participants, including CL patients and healthy volunteers enrolled in the study.

### 2.2 Data and sample collection

The samples were collected from patients referred to the Dermatology Clinic at Mashhad Medical School in Mashhad, and Razi Dermatology Hospital in Tehran IRAN, with suspected CL lesions from January to October 2023. Inclusion criteria for CL patients enrolled in this study were as follows: No history of receiving anti-leishmanial treatment, having an active lesion for a maximum of 1 year, and a characteristic skin lesion-positive PCR for *L. tropica*. The control samples were collected from healthy volunteers undergoing esthetical surgery at Noor Eye Hospital (Tehran). The included volunteers in this study had no history of leishmaniasis or other skin problems.

### 2.3 PCR diagnostics of CL patients

To identify *Leishmania* species, the skin lesion samples, which were collected with a non-invasive and tape disc-based method as described by Taslimi et al. (16), were analyzed with polymerase chain reaction (PCR) assay, targeting internal transcribed spacer 1(ITS1). Then, RFLP analysis using 1U *Hae*III enzyme (Roche) was performed on the PCR products.

### 2.4 Skin biopsies from CL patients and healthy volunteers

A total of 19 CL patients and 10 healthy individuals were included in our study. Demographic characteristics of patients, including age and gender, as well as characteristics of lesions, such as size, number, and duration, were recorded using a patient information sheet. Two-millimeter punch skin biopsies were taken from each included participant. In the CL group, skin biopsies were taken from the border of the lesion. The biopsies were immediately transferred to RNA later solution (Qiagen GmbH, Hilden, Germany) and stored at -20°C for subsequent processing.

### 2.5 RNA extraction, cDNA synthesis, and quantitative real-time PCR

Total RNA of skin biopsy samples was extracted using a total RNA extraction kit (Pars tous, Iran) following the manufacturer’s protocol. RNA quantity was measured using a Nanodrop spectrophotometer (Thermo Scientific, USA), and RNA integrity (RIN) was evaluated on an Agilent Bioanalyzer (Agilent Technologies, USA). Two patient samples were excluded from the study because of low RIN values.

Genomic DNA was removed from the extracted RNA by DNase I (Ambion, USA). After that, cDNA was synthesized using High Capacity cDNA Reverse Transcription Kits (Applied Biosystems, USA) as per the manufacturer’s protocol. qRT-PCR assay was performed in ABI Real-time PCR Detection System (Applied Biosystems, CA, USA) in a reaction volume of 10 μL containing 2X QuantiFast SYBR Green PCR Kit (Qiagen, GmbH, Hilden, Germany) and a specific primer (5pmol). The thermal cycle was as follows: 95°C for 5 min, 40 cycles at 95°C for 20 sec, and 60°C for 30 min.

Additionally, RT-qPCR was performed to evaluate parasite load in each clinical sample using 7SLRNA gene (18). All assays were done in duplicate. The list of primers used in this study is presented in **Table 1**.

**Table 1:** Sequence of primers used in qRT-PCR.

Pathways	Primer name	Forward primer	Reverse primer
**Th17-associated cytokines/receptors**	*il21*	TAGAGACAAACTGTGAGTGGTCA	GGGCATGTTAGTCTGTGTTTCTG
	*il21r*	GGCAAGACCAGTATGAAGAGC	TGACACTGAAAATGTCGTCGG
	*il23a/p19*	ACAGAAGCTCTGCACACTGG	TCCTTTGCAAGCAGAACTGAC
**Th2-associated cytokines/receptors**	*il13*	TCTGCAATGGCAGCATGGTA	GCAAGCTGGAAAACTGCCCA
	*il4*	GCCACCATGAGAAGGACACT	ACTCTGGTTGGCTTCCTTCA
	*il10*	TGATGCCCCAAGCTGAGAAC	GAAGAAATCGATGACAGCGCC
	*il24*	ACAGGACCAGAGGGACAAGA	GCTTTGCAGCCTCTGTTGAAA
**Th1-associated cytokines/receptors**	*il12a/p35*	TTGATGAGCTGATGCAGGC	TGACAACGGTTTGGAGGGAC
	*il12b/p40*	TACTCAGTGGAGTGCCAGGA	GGTGGGTCAGGTTTGATGATG
	*il27/p28*	GAGGGAGTTCACAGTCAGCC	GACGCTCCGGGTCAGAGA
	*il27b/ebi3*	GCCCTGCAGTGGAAGGAAA	CAGCCATGCCGAGCCTGTA
	*ifng*	CTAGGCAGCCAACCTAAGCA	TGGCTCAGATTGCAGGCATA
	*ifngr1*	GGATTCCAGTTGTTGCTGCTTTA	TCTTACCACAGAGATCAAGGACT
	*ifngr2*	TTGAGGTGACCCCAGGAGAA	AAGGGCCTTTGACCTGTTGG
**Transcription factors**	*hif1a*	AACGTCGAAAAGAAAAGTCTCG	TCCTCACACGCAAATAGCTGA
	*tbet*	AAGTGGGTGCAGTGTGGAAA	TGGAGCACAATCATCTGGGT
	*gata3*	TCATTAAGCCCAAGCGAAGG	GTCCCCATTGGCATTCCTC
**JAK-STAT genes**	*jak1*	GCTCCAAATCGCACCATCAC	CAGTGAGCTGGCATCAAGGA
	*jak2*	TACCTCTTTGCTCAGTGGCG	GCCAGTGGGGTTTGATCGT
	*jak3*	CAGTCTCAAGGAGCAGGGTG	TAGGCAGGCCTTGTAGCTGA
	*tyk2*	GAACCGGCTGTGTACCGTT	ACGTCATTCACAAACTCATGCTT
	*stat1*	ATCAGGCTCAGTCGGGGAATA	TGGTCTCGTGTTCTCTGTTCT
	*stat2*	CGACCAGAGCCATTGGAGG	GCAGCTTCCTGCCAGTTCT
	*stat3*	ATCACGCCTTCTACAGACTGC	CATCCTGGAGATTCTCTACCACT
	*stat4*	TCAAGTTTTTGGAGCAGGTGG	TAGAAGCTGCCTCCCAGTCT
	*stat5*	GGCCCCACCAGGTGAAC	GCTCTGTCCTGGGGATTGTC
	*stat6*	TTAAGACAGGCTTGCGGAGG	AGCTGTGCAGAGACACTTGG
**Immune checkpoint genes**	*ctla4*	TGAAGACCTGAACACCGCTC	GGCCACGTGCATTGCTTTG
	*cd80*	GATTGTCATCAGCCCTGCCT	AGTGAGAAAGACCAGCCAGC
	*cd86*	GACGCGGCTTTTATCTTCACC	TCCCTCTCCATTGTGTTGGTT
	*pd1*	ACGAGGGACAATAGGAGCCA	CATACTCCGTCTGCTCAGGG
	*pdl1*	CAGGGCATTCCAGAAAGATGAG	AGACAATTAGTGCAGCCAGGTC
**Extracellular matrix-related genes**	*timp4*	CACTCGGCACTTGTGATTCG	AGGGAAGAGTCAAAAGGCGT
	*mmp9*	CGCAGACATCGTCATCCAGT	AACCGAGTTGGAACCACGAC
**Guanylate-binding protein**	*gbp4*	CCGGCCTACAAATGACAAGC	AGCCGCTTTCCAGTGACAAT
	*gbp5*	TCCTCGGATTATTGCTCGGC	TTTCCGAGGCTCCCGATAGT
**immunoglobulin-like cell surface receptor**	*sirpg*	CCATCCTCCTGGTCCTTTCC	CAGTGCAGAGTGGCTGTCTT
**Epidermal barrier-associated gene**	*flg*	ATTTCGGCAAATCCTGAAGAATCCA	GATGACTGTGCTTTCTGTGCT
**Reference gene**	*gapdh*	GGAGCGAGATCCCTCCAAAAT	GGCTGTTGTCATACTTCTCATGG
***Leishmania* gene involved in intracellular protein translocation**	*7SL RNA*	TGCTGCGTT GACGTGGTGCTC TG	TTGGCTGTGTGTCGGTGTGGCCTGC

### 2.6 Statistical analysis

The relative gene expression analysis was performed using the 2-^ΔΔCT^ method. Human GAPDH was used as an internal control in this analysis. The expression level of the studied genes between CL and control groups was compared by the Mann–Whitney U test and a *p-*value of <0.05 was considered statistically significant. The comparison of differential gene expression was carried out based on the criteria of log2|fold change (FC) |> 1. The absolute quantification was performed by standard curves which were constructed by serial dilution of cDNA products of 1.5 × 10^6^ *L. tropica*. GraphPad Prism 8.0 (GraphPad Software Inc., CA, USA) software was used for statistical analyses and drawing scatter plots. The volcano plot was created using ggplot2 (v 3.3.5) and ggrepel (v 0.9.1) packages in R (4.1.2, https://www.r-project.org/) (19). Principal component analysis (PCA) plot was generated using tidyverse package. Pearson correlation test with the R corrplot package (v 4.4.0) was applied to identify correlations between the studied genes. Absolute correlation values ≥ 0.8 were considered strong correlation. The correlation matrix was visualized with the R corrplot package (v 0.92).

## 3 Results

### 3.1 Clinical characteristics and parasite load in CL lesions

This study included 17 *L. tropica-*infected CL patients with an average age of 42.88 ± 4.5 years old, including 12 (70.6%) female and 5 (29.4%) male. Ten healthy individuals, including 7 (70%) females and 3 (30%) males with an average age of 50.7± 2.97 years old, were also involved in this study. Eight patients (41.2%) had a single lesion and the rest of them were found with more than two skin lesions at the time of enrollment. The average duration and surface area of lesions in the patients were 26.15± 7.9 (cm^2^) and 4.15± 0.63 (month), respectively. The present study investigated the parasite load in lesions of patients infected with *L. tropica*. The average load of parasites found in the lesions was 7700 ± 3700 parasites/cells, with a range from 1400 to 17100 parasites/cells. Further, the analysis revealed no significant correlation between the characteristics of the lesions and the parasite load in each patient. The results of the parasite load and lesion characteristics are shown in **Supplementary Figure 1**.

### 3.2 Differential gene expression analysis

**Figure 1:**
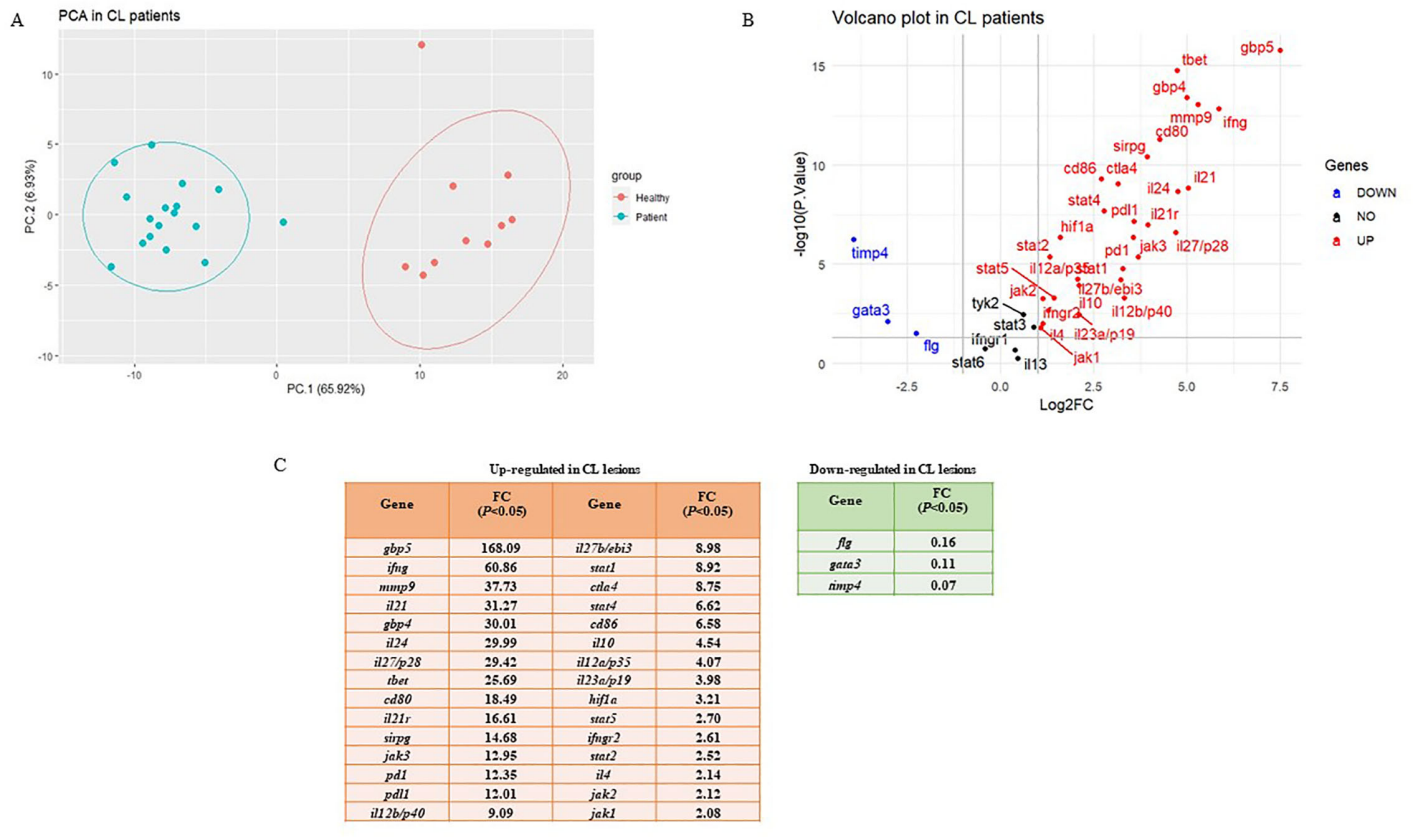
Differential expression gene analysis. **(A)** Principal Component Analysis (PCA) for skin lesions of CL patients (blue circles, n = 17) compared to the healthy group (red circles, n = 10). **(B)** Volcano plots of the tested genes in CL patients when compared to a healthy group. **(C)** Statistically significant up-and down-regulated genes in CL lesions compared to healthy skin. CL, cutaneous leishmaniasis; FC, fold change.

To determine the differentially expressed genes, the magnitude of fold changes among 38 immune-related genes in the skin lesions of *L. tropica-*infected patients to healthy group were compared, and up-and down-regulated genes were determined. As depicted in **Figure 1A**, principal component analysis (PCA) on gene expression of the studied genes separated patients from healthy controls across the PC-1 of 65.92%.

The list of genes included in the study is as follows: 11 Th1/Th2/Th17- associated cytokines (*ifng, il12a/p35, il12b/p40, il27b/ebi3, il27/p28, il23a/p19, il4, il13, il10, il24*, and *il21*), three cytokine receptors (*il21r, ifngr1*, and *ifngr2*), five immune checkpoints (*pd1, pdl1, ctla4*, *cd80*, and *cd86*), 10 Janus kinase/signal transducer and activator of transcription (JAK-STAT; *stat1*, *stat2, stat3, stat4, stat5, stat6, jak1, jak2, jak3* and *tyk2*), two extracellular matrix related genes (*mmp9* and *timp4*), two guanylate-binding protein (*gbp4* and *gbp5*), three transcription factors (TFs; *tbet, gata3*, and *hif1a*), one immunoglobulin-like cell surface receptor (*sirpg*), and one epidermal barrier function associated gene (*flg*). Further, a volcano plot of Log2 fold changes versus the *P* values (-Log10) of the studied genes depicted the identified statistically significant up-and-down-regulated genes in CL patients compared to the control (**Figure 1B**). The fold change values are also presented in **Figure 1C**.

### 3.3 Intralesional expression level of Th1/Th2/Th17-associated cytokines and their receptors in *L. tropica*-infected patients

**Figure 2:**
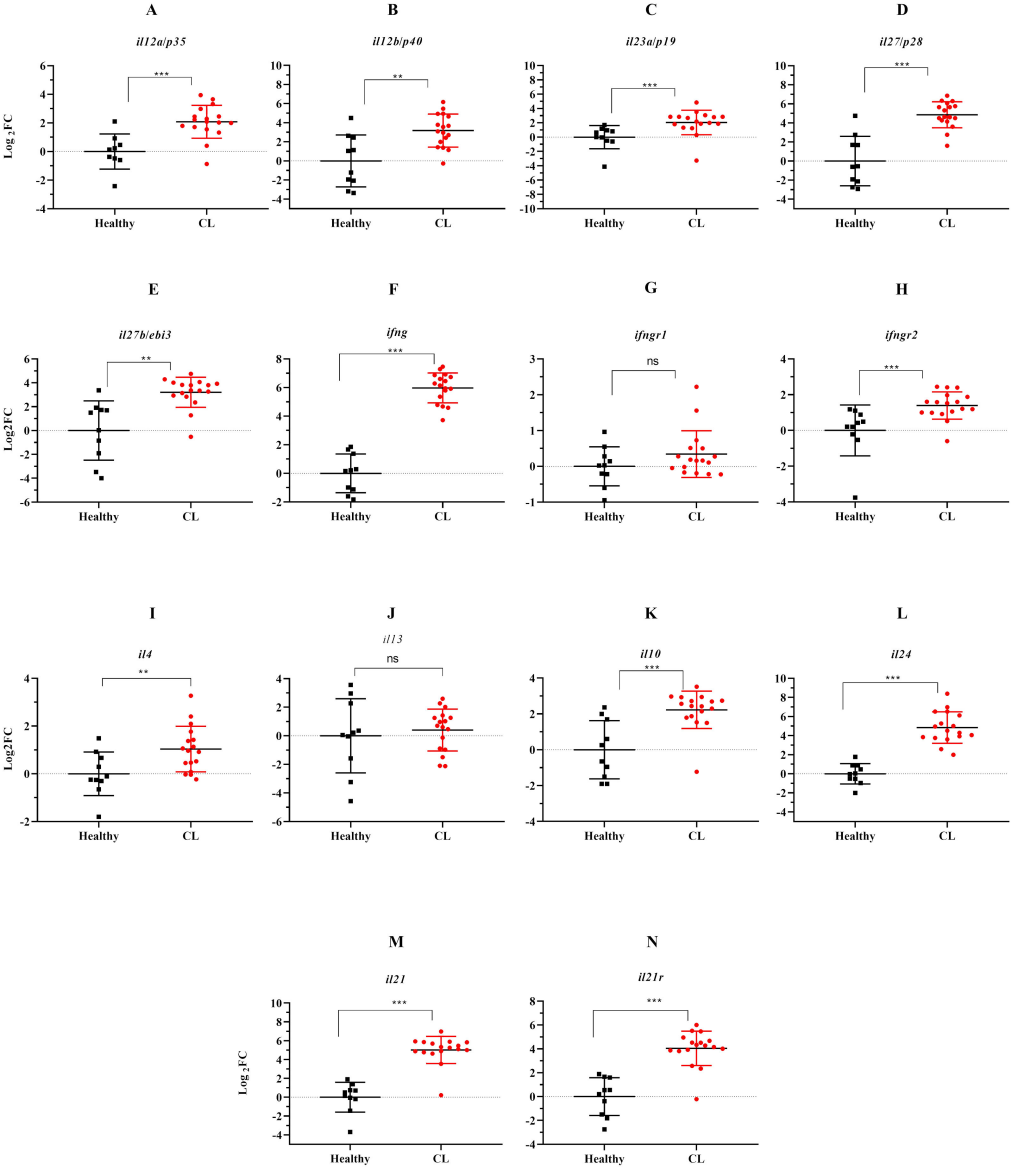
The effect of *L. tropica* infection on mRNA expression of 11 cytokine genes in the skin lesion of CL patients. Relative mRNA expression of **(A)** *il12a/p35*, **(B)** *il12b/p40*, **(C)** *il23a/p19*, **(D)** *il27b/p28*, **(E)** *il27b/ebi3*, **(F)** *ifng*, **(G)** *ifngr1*, **(H)** *ifngr2*, **(I)** *il4*, **(J)** *il13*, **(K)** *il10*, **(L)** *il24*, **(M)** *il21*, and **(N)** *il21r*. Data are normalized using *gapdh* as the control gene. **P < 0.01, and ***P< 0.001. The results were shown as the mean +/- SD of duplicate measurements. CL, Cutaneous leishmaniasis. ns, non-significant.

We evaluated the expression of several Th1/Th2/Th17-associated cytokines and corresponding receptors by the qRT-PCR method. Compared to the healthy group, expression levels of *il12a/p35, il12b/p40, il27b/ebi3, il27/p28*, and *il23a/p19*, as members of *il12* family cytokines showed increased expression levels in *L. tropica*-infected patients (**Figures 2A–E**). All up-regulated genes were statistically significant (*P*<0.05). In addition to *il12a* and *il12b*, the expression of other Th1-related cytokines/cytokine receptors, including *ifng, ifngr1*, and *ifngr2*, were also evaluated, and *ifng* and *ifngr2* found as statistically significantly over-expressed genes, while the expression of *ifngr1* was decreased (*P*>0.05), in *L. tropica*-infected patients (**Figures 2F–H**). Further, assessing the expression of Th2-associated cytokines/cytokine receptors, including *il4, il13, il10*, and *il24*, showed statistically significant up-regulation in patients versus the healthy group (**Figures 2I–L**). *il21* and its corresponding receptor, *il21r*, were a pair of genes upregulated as members of the Th17 cell subset. Besides, we found the expression magnitude of Th1-associated cytokines was higher than Th2-associated cytokines.

### 3.4 Expression levels of *jak-stat* genes and other Th1/Th2-related transcription factors during *L. tropica* infection

**Figure 3:**
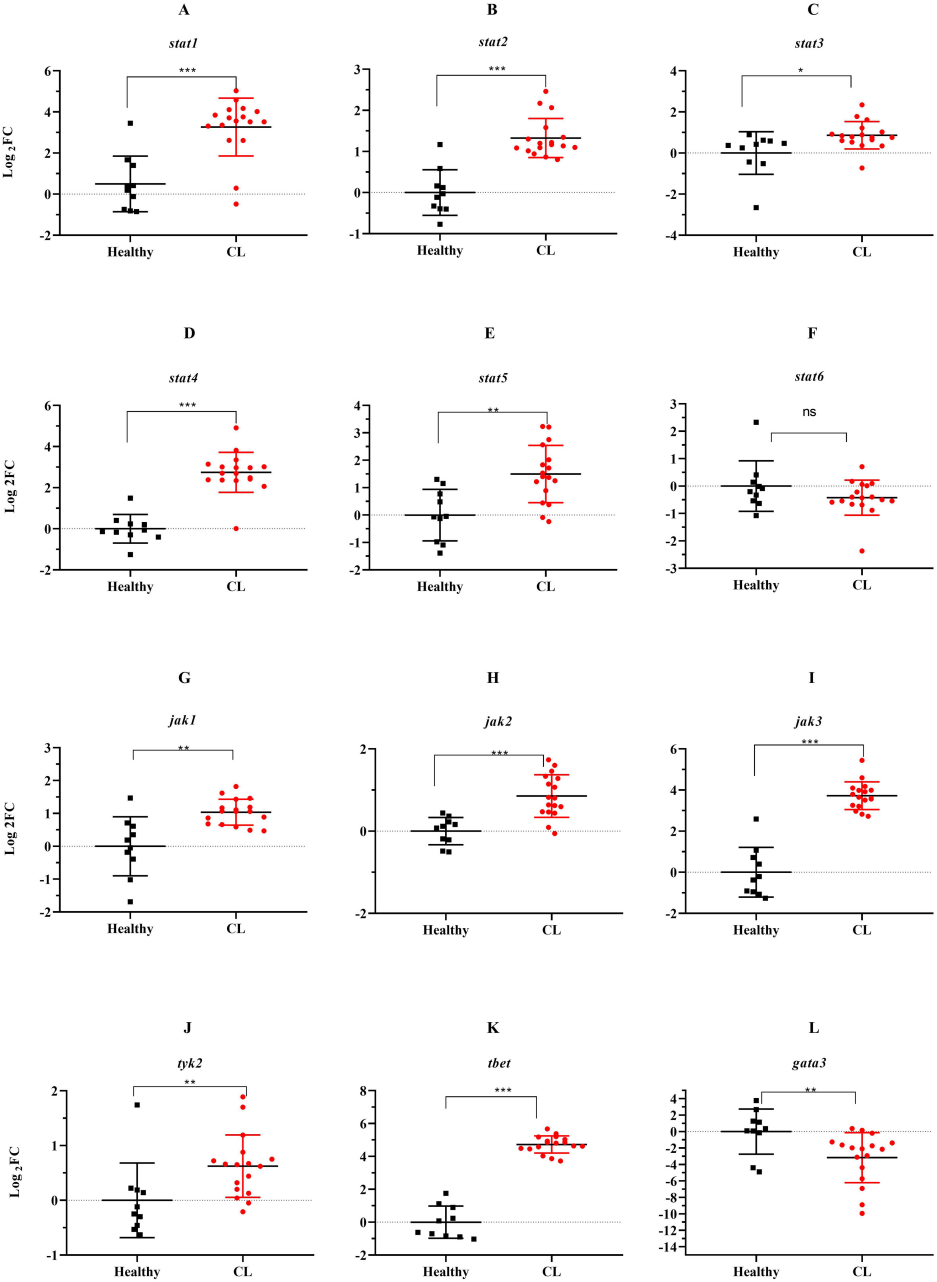
The expression levels of 10 *jak-stat*s genes and two Th1/2 related-TF between *L. tropica* infected patients and healthy group. **(A)** *stat1*, **(B)** *stat2*, **(C)** *stat3*, **(D)** *stat4*, **(E)** *stat5*, **(F)** *stat6*, **(G)** *jak1*, **(H)** *jak2*, **(I)** *jak3*, **(J)** *tyk2*, **(K)** *tbet*, and **(L)** *gata3*. Data are normalized using *gapdh* as the control gene. *P<0.05, **P < 0.01, and ***P< 0.001. The results were shown as the mean +/- SD of duplicate measurements. CL, Cutaneous leishmaniasis.

Next, we assessed expression levels of *jak-stat* genes and other Th1/Th2-related transcription factors in the lesion of *L. tropica* patients. The relative expression analysis of six *stat* genes, namely *stat1, stat2, stat3, stat4, stat5*, and *stat6*, in *L. tropica*-infected patients, revealed all *stat* genes except for *stat6* were upregulated (**Figures 3A–E**). However, we observed down-regulation in *stat6* expression level; albeit not statistically significant (**Figure 3F**). Among *jak* family genes, *jak1-3* genes were found to be statistically significantly over-expressed (*P*<0.001) (**Figures 3G–I**), while no change was observed in the expression of the *tyk2* gene (**Figure 3J**). Additionally, the expression of tbet and *gata3*, as key TFs linked to Th1 and Th2 cell differentiation, was significantly upregulated (**Figures 3K, L**).

### 3.5 Differential expression of the immune checkpoints and other genes in the lesions of *L. tropica* infected patients

**Figure 4:**
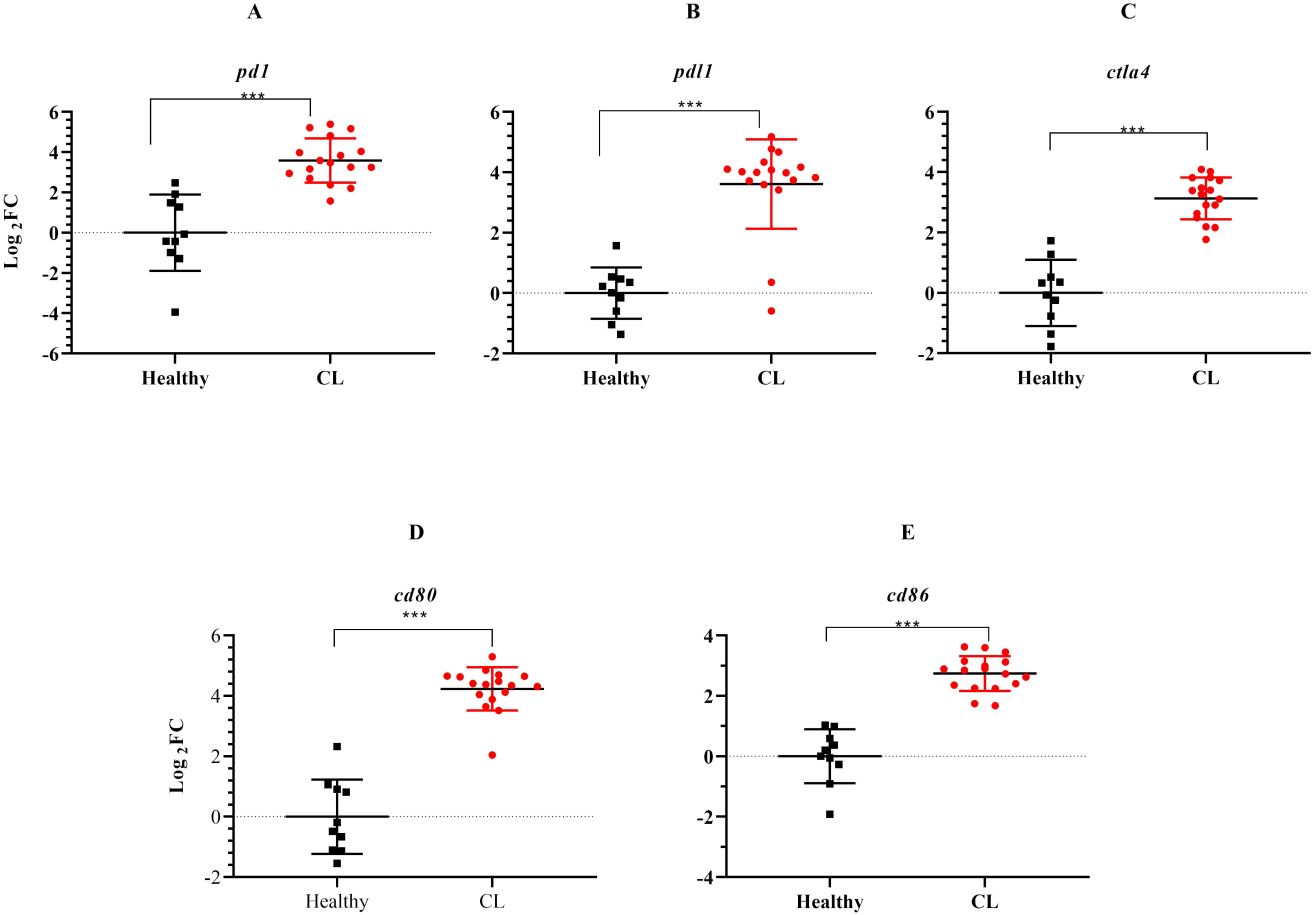
The expression levels of immune checkpoint genes between *L. tropica* infected patients and healthy group. **(A)** *pd1*, **(B)** *pdl1*, **(C)** *ctla4*, **(D)** *cd80*, **(E)** *cd86*. Data are normalized using *gapdh* as the control gene. ***P< 0.001. The results were shown as the mean +/- SD of duplicate measurements. CL, Cutaneous leishmaniasis.

**Figure 5:**
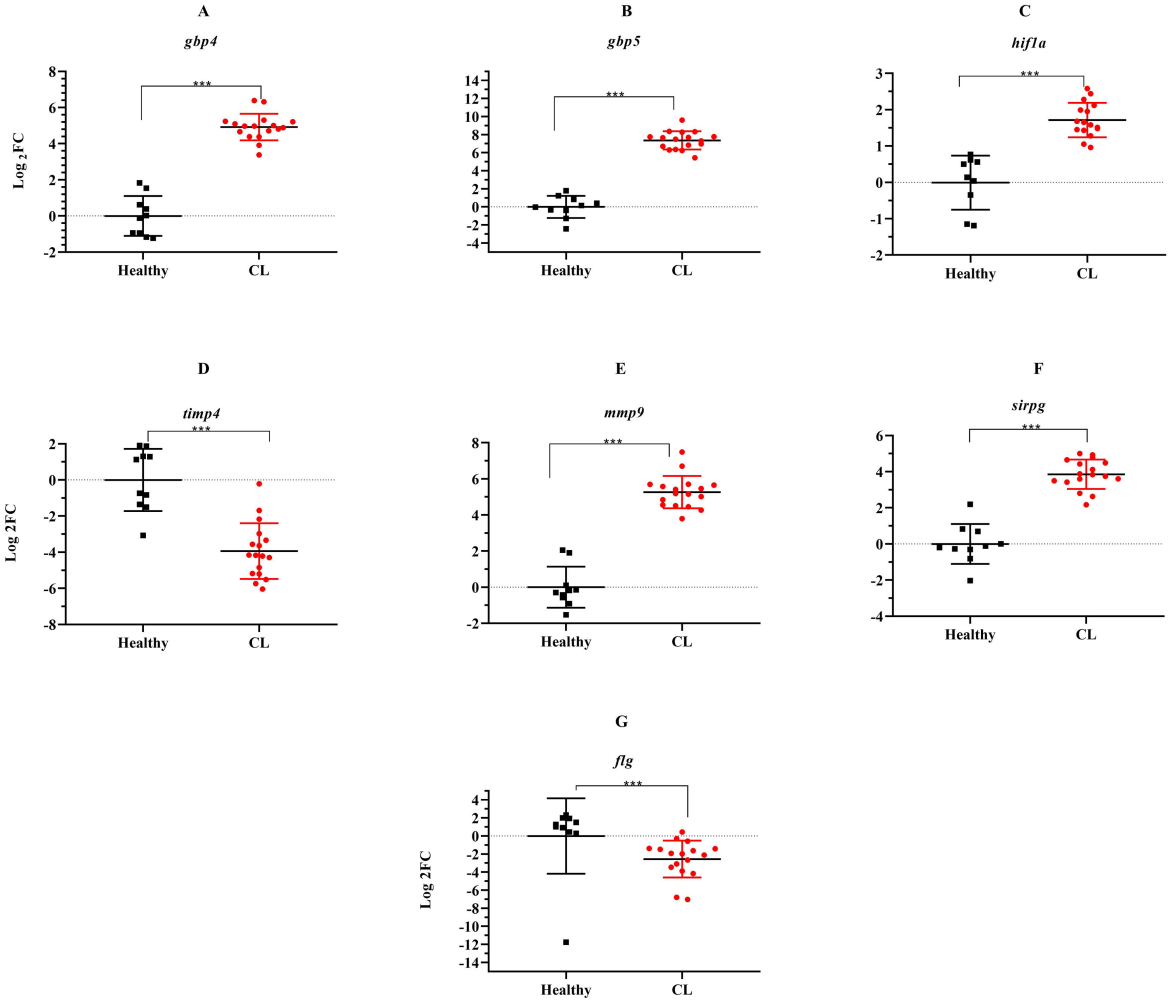
The relative expression of several genes induced during *L. tropica* infection. **(A)** *gbp4*, **(B)** *gbp5*, **(C)** *hif1a*, **(D)** *timp4*, **(E)** *mmp9*, **(F)** *sirpg*, and **(G)** *flg*. Data are normalized using *gapdh* as the control gene. ***P< 0.001. The results were shown as the mean +/- SD of duplicate measurements CL, Cutaneous leishmaniasis.

During the course of an immune response, various immune checkpoint pathways undergo activation or deactivation. Inhibitory immune checkpoint molecules, including CTLA4/CD80/CD86 and PD1/PDL1, are known to suppress T-cell activation. Our results herein showed that the expression level of inhibitory immune checkpoint molecules, including *ctla4/cd80/cd86* and *pd1/pdl1* genes was heightened in the lesions of *L. tropica*-infected patients compared to the skin of healthy individuals (**Figures 4A–E**). The expression level of seven other genes, which functions as transcription factor (*hif1a*), extracellular matrix (*mmp9, timp4*), interferon-inducible GTPases (*gbp4, gbp5*), T-cell adhesion (*sirpg*), and epidermal barrier (*flg*) was also assessed by qRT-PCR. As depicted in **Figure 5**, expression of *gbp4, gbp5, hif1a, mmp9*, and *sirpg* was increased in the patients compared to the healthy group (*P*< 0.001). Whereas expression of *timp4* and *flg* genes was found to be downregulated (*P*< 0.001).

### 3.6 Correlation analysis

**Figure 6:**
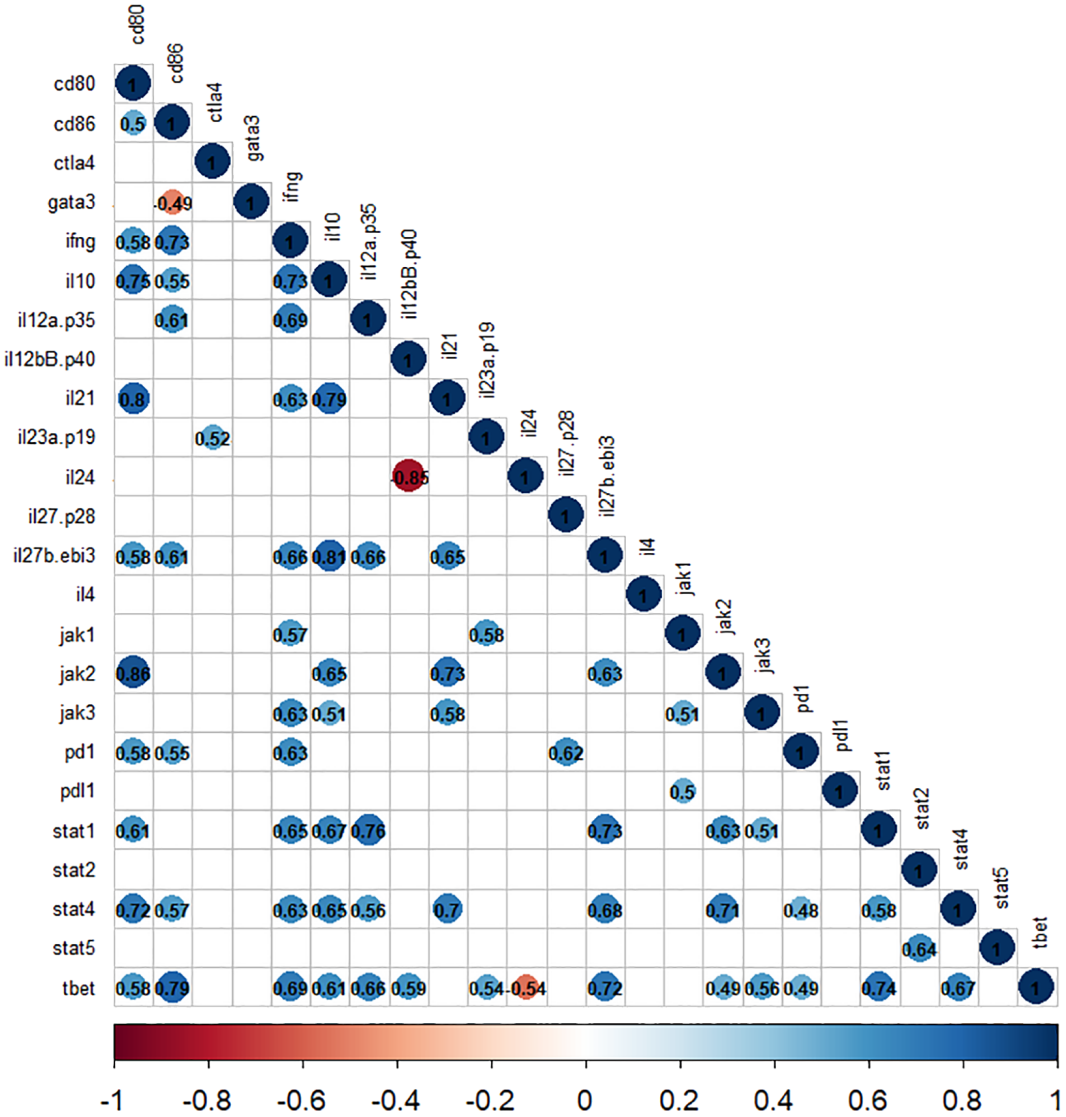
The correlation matrix shows the relationships between cytokines and *jak-stat* genes in *L. tropica*-infected patients. The correlation values are displayed in matrices and color-coded: blue for positive correlations and red for negative correlations. Any correlation values that are not statistically significant (*P*>0.05) are represented by blank white spaces.

The correlation analyses demonstrate a significant positive correlation between the expression of cytokine and *jak-stat* genes. Specifically, the expression of *ifng, il12(p35), and il35(ebi3)* showed a positive correlation with *stat1* and *stat4* expression (r2>0.5, *P*<0.05). Moreover, within *jak* genes, the expression of *jak1* was associated with *ifng*, and *il23(p19)*, while *jak2* was linked to *il10*, *il35(ebi3)*, and *il21* (r2>0.6, *P*<0.05). Furthermore, the *jak3* gene expression exhibits a correlation with *ifng, il10*, and *il21* (r2>0.5, *P*<0.05), as depicted in **Figure 6**. Additionally, our analysis highlighted a positive correlation between *cd80* and *stat1* (r2>0.6, *P*<0.05), as well as between *cd86* and *stat4 (r2>0.5, P<0.05)* among immune checkpoint genes. Notably, the correlation values for the other selected genes are shown in **Figure 6**.

## 4 Discussion

Previous studies have shown the critical role played by cytokine expression status in determining the ultimate fate of leishmaniasis, favoring either the host or the parasite (20, 21). The aberrant activation of JAK-STATs signaling pathway is recognized as the main factor behind regulation mediated by these cellular mediators in various inflammatory and infectious diseases (22). However, signaling pathways are complex and interconnected, the central regulatory role of the pathway holds promise for potential interventional implications in a multitude of diseases (14, 23). Herein, we investigated the intralesional expression of 24 cytokine/*jak*/*stat* genes in the skin lesions of *L. tropica*-infected patients. We have also evaluated the expression of 5 inhibitory immune checkpoint genes along with nine genes related to transcription factors, extracellular matrix, interferon-inducible GTPases, T-cell adhesion, and epidermal barrier function in the lesions of *L. tropica*-infected patients.

We observed that the expression of several pro-inflammatory cytokines/cytokine receptors, including *il12* family, *ifng*, and *ifngr2*, were increased in the skin lesion of *L. tropica*-infected patients. IL-12 is considered a crucial host-protective cytokine in CL by driving the development of IFN-γ -producing T cells (24, 25). The regulatory effect of IL-12 on T-cells is achieved through the transduction of JAK2/TYK2 and STAT4 pathways (26). The biological effect of IFN-γ, as another crucial pro-inflammatory cytokine in CL (27), is mediated by the activation of JAK1/JAK2 and STAT1 in T cells. The signaling of IL-12 and IFN-γ through STAT4 and STAT1 also stimulated the expression of Th1 master regulator gene, Tbet, and induced further IFN-γ production (28). Our data showed that in addition to *il12* (*p35* and *p40*) and *ifng*, the expression of *jak1*, *jak2*, *stat1*, *stat4*, and *tbet* was also increased in the skin lesion of *L. tropica* patients. The results may reflect the effective recruitment of immune cells characterized by increased pro-inflammatory cytokines and activated downstream genes. In agreement with our result, skin samples of CL patients infected with *L. braziliensis* showed upregulation of *ifng* and *stat1*. The study also revealed that high levels of *ifng* expression in the lesions were associated with the activation of cytolytic pathways (29). In other studies, blood (26) and skin samples (*manuscript in preparation*) of patients with CL due to *L. tropica* demonstrated overexpression of *stat1* and IFN-responsive genes. Nonetheless, downregulation of several immune-related genes, including *stat1*, *nfκb*, *ifngr2*, and *il12rb2* were reported in the blood of the severe clinical form of CL, diffuse CL caused by *L. mexicana* (30). Further, *gbp* genes, which induced in response to IFN, reported to play a role in conferring cell-autonomous immunity against various pathogens (29, 31, 32). However, the role of these genes in CL is not well established. Previous reports on overexpression of different isoforms of the GBPs family in CL patients suggested a possible role for these large GTPases in protection against *Leishmania* infection (33–35). For instance, an overexpressed level of *gbp5* was reported in *L. braziliensis* and *L. tropica* CL biopsy samples, with involvement in inflammation (33, 35). Similarly, we herein found overexpression of *gbp4* and *gbp5* in *L. tropica* lesions. This expression pattern may show the predominant transcriptional alternation linked to *ifng* in CL due to *L. tropica*.

We could also show the upregulation of *il23* (*p19/p40*) and *il27* (*p28/ebi3*) as other members of the *il12* family cytokine. The increased level of IL-23 is described to have an association with the maintenance of Th17 cells, which may contribute to the persistence of inflammation or promote the chronicity of CL infection caused by *L. amazonensis* and *L. braziliensis* (36). By contrast, the correlation of *il23* expression with healed *L. major* CL lesions and reducing disease immunopathology has been documented (37). IL-23 exerts its function mainly via activation of JAK2/TYK2 and STAT3 (38). Notably, STAT3 and TYK2 are described as a crucial component for Th17 cell development and the key isoform of the JAK family in the IL-23 signaling cascade, respectively (39). However, in our study, the *il23*-dependent effects were not significantly changed in the lesion of *L. tropica*-infected patients. On the other hand, IL-27 (P28/Ebi3), is acknowledged to have a dual function in inducing the immuno-pathology of *Leishmaniasis* (40–42). The dubious role of IL-27, on one side, favors the host by induction of protective immune response in *L. major* infection (40), an effiect mainly mediated inhibition of Th2 cell response and, on the other side, contributes to enhancing disease during *L. amazonensis* and *L. braziliensis* infection through IL-10 induction (41, 43). The specific effects of IL-27 in immune cell subsets occurred after activation of JAK1/JAK2 and mainly three isoforms of STAT, namely, STAT1, STAT3, and STAT5. In a STAT1-dependent manner, the signaling molecule can positively affect the expression of T-bet and IFN-γ as well as induce downregulation of the GATA3 and the Th2-associated cytokines (44, 45). Based on the observed expression pattern in our study, we speculated that the induction of *il27* in *L. tropica* CL lesions may have an immunostimulatory effect, presumably favoring the host immunity through downregulation of Th2 type genes. Besides, IL-27 as well as IFN-γ is reported to have the potential to stimulate overexpression of co-inhibitory checkpoints and their key cytokine, IL-10, on T cells in a STAT1-dependent manner, which mitigates tissue damage (46, 47). In the present study, evaluating the expression of two inhibitory receptors (*pd1, ctla4*) and their corresponding ligands (*pdl1, cd80, cd86*) as well as *il10* showed overexpression in the lesion tissue of CL patients. Aligned with our result, a recent study highlighted an increase in the expression of *pdl1* in skin lesions of CL patients infected by *L. donovani* (48). However, the exact role of immune checkpoints during CL infection is still inconclusive. In our study, these genes seem to have a role in maintaining immune homeostasis which prevents overwhelming inflammation. Besides, we found expression of downstream genes regulated by *il10* namely, *tyk2, jak1*, and *stat3* remained unchanged except for *jak1* in the CL skin lesions. IL-10 has been reported to counteract an exacerbated immunopathology induced by a high magnitude of Th1 immune response and accelerate the wound healing process, at the same time, playing a role in the progression of the infection and parasite persistence in CL (49–51). Further research is needed to fully elucidate the mechanisms by which IL-10 affects the course of the disease and to determine the optimal dosage and timing of IL-10 administration in CL patients.

Alongside IL-10, IL-35 (EBI3/p35), as regulatory T and B cell-derived cytokine (52, 53), also has immunosuppressive activity. A recent study reported a significant increase in IL-35 production in PBMC infected by *L. infantum* and *L. donovani*, whereas no change in IL-35 concentrations was identified in response to *L. major* and *L. tropica* infection (54). We could also herein identify the overexpression of *ebi3* and *p35* in the lesion of *L. tropica-*infected patients. Additionally, evaluating *jak/stat* genes transduced by IL-35 showed the overexpression of *jak1, jak2, stat1*, and *stat4*. This pattern indicates that the simultaneous expression of immunosuppressive cytokine genes like *il10* and *il35* in *L. tropica*-infected tissues possibly resulted in the persistence of the lesions despite the presence of a high magnitude of inflammatory cytokine. However, further investigation is warranted to elucidate the extent of involvement of these cytokines in the immune response against CL caused by *L. tropica*.

IL-24 is a multifunctional cytokine produced by both immune and non-immune cells, such as keratinocytes (55) and its biological activity is mediated via the JAK1/STAT3 pathway. Upregulation of IL-24 is reported to be involved in wound repair and pathogenesis of inflammatory and autoimmune diseases such as psoriasis (56–58). However, the protective role of IL-24 has been described in animal studies of several diseases such as inflammatory bowel disease (59). In the context of leishmaniasis, the biological role and expression pattern of IL-24 is poorly studied, with one preprint report showing elevated levels of IL-24 in CD274+ and IDO1+ myeloid cells in human CL skin lesions (60). Overall, based on the role of *il24* in wound healing, we tend to speculate a protective role against *L. tropica* infection by modulating immunopathology following increased production of inflammatory response. However, further studies need to ascertain the exact role of this cytokine in CL.

We could also document the upregulation of *il21*/*il21r* and its downstream *jak1*/*jak3* and *stat1* genes. The activation of STAT1 by IL-21 is noted to intensify Th1 immune response and IFN-γ production (61, 62). Therefore, the high level of *il21/il21r* expression in our study can be considered another possible factor for the significantly elevated level of *ifng* and its downstream genes in *L. tropica* CL lesion. IL-21 plays an important role in the disease pathology of various diseases by increasing the T-cell effector responses (63). However, studies related to the role played by this cytokine in the context of leishmaniasis are limited (43, 55, 64–66), the current evidence suggests a potential disease-enhancing role for this mediator.

**Figure 7:**
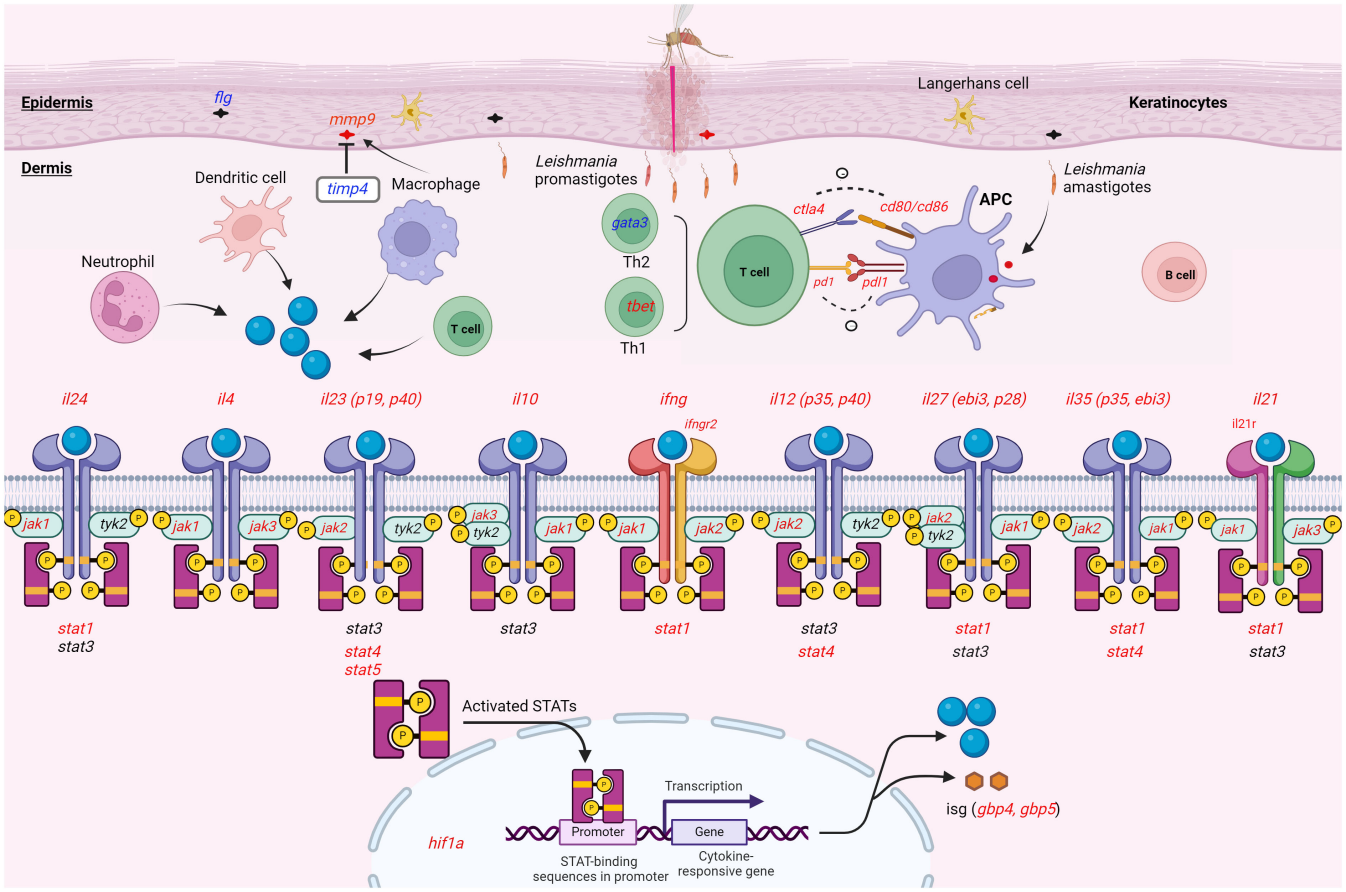
Schematic representation illustrating the expression pattern of the genes analyzed within the lesion of CL patients infected with *L. tropica*. This figure provides a whole overview of the signaling cascade without linking the effect of the cytokines to a specific immune cell. Upon *Leishmania* entry into the dermis, different innate cells such as neutrophils, macrophages, and dendritic cells infiltrate the site of infection. The cell produces effector molecules like cytokine resulting in either a permissive or hostile environment for the parasite. The effector function of the immune cell also instructs T helper (e.g., *tbet*/*gata3*) cell differentiation. In skin resident cells as well as migratory immune cells, the Janus kinase (JAK)-signal transducer and activator of transcription (STAT) pathway can be induced by a myriad of cytokines present during skin inflammation. Cytokines (*il24, il4, il10, ifng, il12, il27, il35* and *il21*) interact with the corresponding receptors (*ifngr2*, and *il21r*), which phosphorylate JAKs (*jak1, jak2, jak3* and *tyk2*) and activates *stat* genes (*stat1-6*). Activated STAT forms a dimer. The dimerized STAT translocate to the nucleus and mediates the transcriptional regulation of target genes like interferon-stimulated gene (ISG; *gbp4*, and *gbp5*), and inflammatory molecules. During an immune response, different stimulatory and inhibitory immune checkpoints (*pd1/pdl1*, *ctl4/cd80/cd86*) pathways can also induce or suppress. This interaction mainly occurs between a T cell and a macrophage, and DC as antigen-presenting cells (APCs). Up- and down-regulated genes were presented in red and blue colors, respectively. Gene with an unchanged expression was also shown in black. CL, cutaneous leishmaniasis. This image was created with BioRender.com.

The effect of GATA3 expression change on FLG, which encodes the epidermal barrier protein filaggrin, in keratinocytes was pointed out in human inflammatory skin diseases (67). Our data showed that the expression of both genes was downregulated in the lesion of *L. tropica* infection. It can be reasonable to speculate that the downregulation of *flg* was influenced by the downregulation of *gata3* and this downregulation affected the epidermal integrity of the skin and skin microbiome composition in *L.tropica* CL lesions. Along with this gene, we found the overexpression level of *mmp9* and the downregulation of its natural regulator, *timp4*, in the CL lesions. An imbalanced production of MMPs and TIMPs is reported in the occurrence of tissue damage in various diseases (68–70). Our findings suggest that downregulated *timp4* may augment *mmp9* activity and the imbalanced level of *mmp9* and *timp4* could affect the resolution of the *L. tropica* CL lesion formation. Notably, the overall findings of this study are summarized in **Figure 7**, illustrating the conceptual framework of cytokine-receptor engagement activating the JAK-STAT pathway in the inflammatory and immune response activation observed in the lesions.

One of the limitations of our study is sole focus on transcriptome of the studied genes. Analysis of protein expression of the studied genes and integration of transcript and protein data in a larger cohort would further strengthen these findings, and may significantly enhance our understanding of signaling pathways involved in *L. tropica* infection.

In summary, our study herein suggests the dysregulation of 11 pro- and anti-inflammatory cytokines and their associated *jak-stat* genes in the skin lesion of CL patients infected by *L. tropica*. We also highlighted the expression of *il21, il24* and *il35* genes, which are rather understudied in human CL (**Figure 7**). A significant positive correlation between stat1 and stat4 expression and the expression of various cytokines and immune checkpoint genes was observed in patients infected with *L. tropica*, indicating potential interconnections between these factors in the context of the infection. Furthermore, we detected elevated levels of Th1 and Th2 response in the lesions of patients infected with *L. tropica*. This suggests the balance of cytokines is a crucial factor in determining the clinical outcome, rather than the quantity of cytokines. Our findings warrant further exploration of the regulatory impact and the functional significance of the cytokines and their downstream genes on the pathogenesis of CL on larger cohorts. Such an investigation is likely to yield valuable insights into their biological functions, potentially paving the way for the identification of novel therapeutic options to counter CL.

## Data Availability

The original contributions presented in the study are included in the article/**Supplementary Material**. Further inquiries can be directed to the corresponding authors.
