# Supplementary material for: Intralesional gene expression profile of JAK-STAT signaling pathway and associated cytokines in Leishmania tropica-infected patients
Source: Front Immunol. 2024 Sep 19;15:1436029. doi: 10.3389/fimmu.2024.1436029 (PMC11446769; doi:10.3389/fimmu.2024.1436029)

Supplementary File 1. Clinical characteristics and parasite load in CL lesions.

Patients number	Target Name	Parasites/Cell	Gender	Age	Lesion Duration (Month)	Lersion size of punch biopsy (cm2)	Lesion number
1	7SLRNA	9141.111328	F	44	7	4x5	4
2	7SLRNA	11411.14844	F	25	1	2x2	1
3	7SLRNA	4662.216797	F	67	3	10x7	1
4	7SLRNA	6206.295898	F	50	4	1x1	3
5	7SLRNA	10031.02051	F	21	7	1x1	2
6	7SLRNA	9029.564453	M	29	7	2x3	1
7	7SLRNA	5125.890625	F	56	4	2x3	3
8	7SLRNA	6729.735352	F	27	4	10x7	1
9	7SLRNA	2652.817383	F	58	6	12x7	4
10	7SLRNA	5488.623047	M	18	7	12x7	1
11	7SLRNA	6644.430664	M	67	2	1x1	2
12	7SLRNA	8380.103516	M	60	1	1x1	11
13	7SLRNA	6828.613281	M	23	4	1x1	3
14	7SLRNA	7442.40332	F	64	5	15x7	6
15	7SLRNA	17198.19922	F	30	1	1x1	2
16	7SLRNA	1427.558228	F	60	8	8x6	1
17	7SLRNA	12521.90527	F	29	5	1x1	2

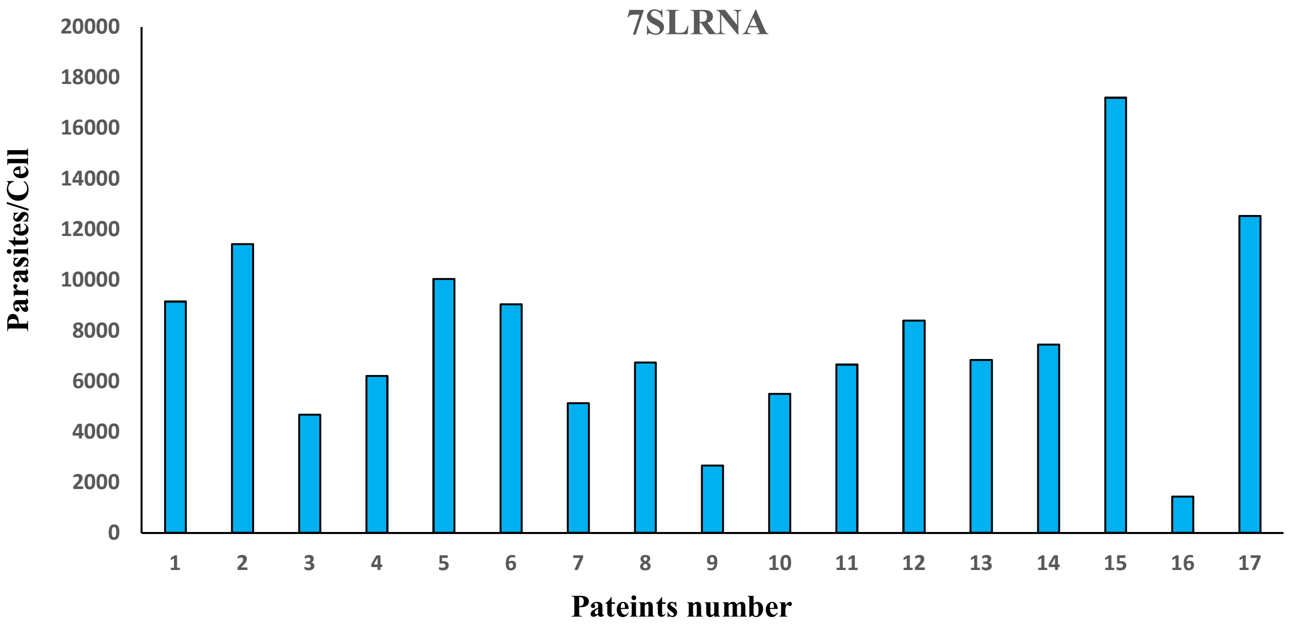

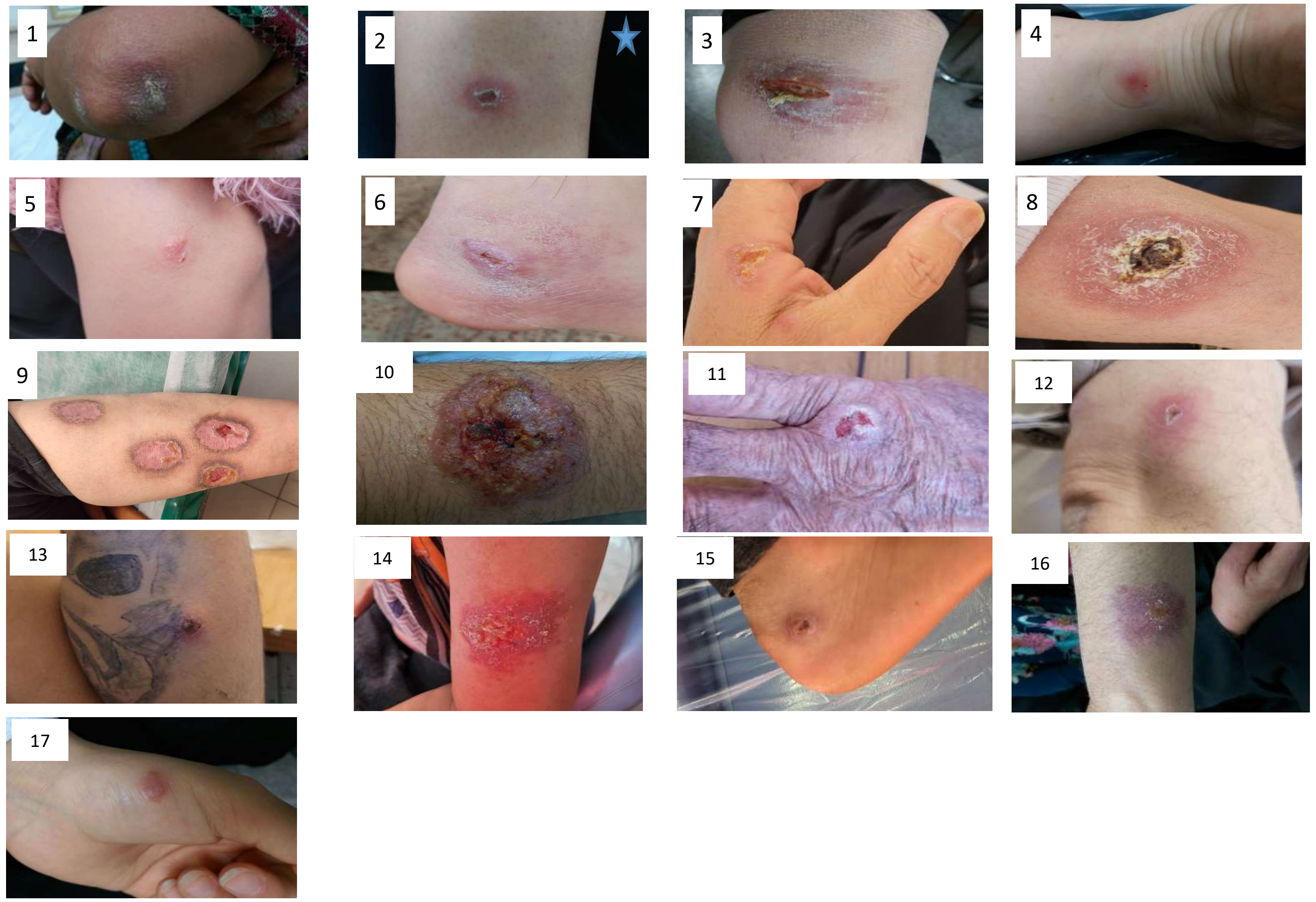

Supplement: Supplementary file 1 [file DataSheet1.pdf]
